# Evaluating the granularity and statistical structure of lesions and behaviour in post-stroke aphasia

**DOI:** 10.1093/braincomms/fcaa062

**Published:** 2020-05-19

**Authors:** Ying Zhao, Ajay D Halai, Matthew A Lambon Ralph

**Affiliations:** MRC Cognition and Brain Sciences Unit, University of Cambridge, Cambridge CB2 7EF, UK

**Keywords:** stroke, aphasia, lesion–symptom mapping, middle cerebral artery, cortical vascular branches

## Abstract

The pursuit of relating the location of neural damage to the pattern of acquired language and general cognitive deficits post-stroke stems back to the 19th century behavioural neurology. While spatial specificity has improved dramatically over time, from the large areas of damage specified by post-mortem investigation to the millimetre precision of modern MRI, there is an underlying issue that is rarely addressed, which relates to the fact that damage to a given area of the brain is not random but constrained by the brain’s vasculature. Accordingly, the aim of this study was to uncover the statistical structure underlying the lesion profile in chronic aphasia post-stroke. By applying varimax-rotated principal component analysis to the lesions of 70 patients with chronic post-stroke aphasia, we identified 17 interpretable clusters, largely reflecting the vascular supply of middle cerebral artery sub-branches and other sources of individual variation in vascular supply as shown in classical angiography studies. This vascular parcellation produced smaller displacement error in simulated lesion–symptom analysis compared with individual voxels and Brodmann regions. A second principal component analysis of the patients’ detailed neuropsychological data revealed a four-factor solution reflecting phonological, semantic, executive-demand and speech fluency abilities. As a preliminary exploration, stepwise regression was used to relate behavioural factor scores to the lesion principal components. Phonological ability was related to two components, which covered the posterior temporal region including the posterior segment of the arcuate fasciculus, and the inferior frontal gyrus. Three components were linked to semantic ability and were located in the white matter underlying the anterior temporal lobe, the supramarginal gyrus and angular gyrus. Executive-demand related to two components covering the dorsal edge of the middle cerebral artery territory, while speech fluency was linked to two components that were located in the middle frontal gyrus, precentral gyrus and subcortical regions (putamen and thalamus). Future studies can explore in formal terms the utility of these principal component analysis-derived lesion components for relating post-stroke lesions and symptoms.

## Teaser

Brain damage post-stroke is not random but is constrained by the neurovascular system. Zhao *et al.* applied principal component analysis to post-stroke lesions to construct a parcellation of the middle cerebral artery cortical supply. This parcellation was used in lesion–symptom mapping, and mis-localization analyses showed lower error compared to Brodmann areas.

## Graphical Abstract

**Graphical Abstract:**
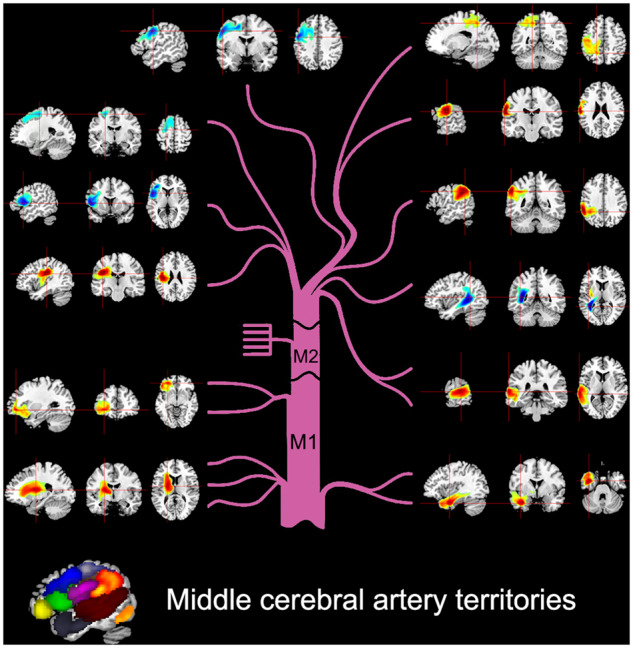

## Introduction

Classically, investigators used post-mortem dissections to provide insight into which brain areas were related to different behaviours/functions. These included the seminal studies of Broca and Wernicke who identified areas of the brain related to speech production and comprehension, respectively (Broca, 1861; Wernicke, 1874). A subsequent development allowed the general area of damage to be explored, *in vivo*: one consequence of the tragic World Wars was that, for soldiers who survived missile head injuries, the trajectory of the missile could be determined (by entry/exit points). This allowed researchers to infer the location of damage and determine the effects on behaviour in greater detail (e.g. Head, 1926; Schiller, 1947; Tallal and Newcombe, 1978). Contemporary *in vivo* lesion mapping became possible with the invention of computerized axial tomography scan (CAT scan) (Ambrose and Hounsfield, 1973) and MRI (Mansfield and Maudsley, 1977). MRI scanners can now image tissue with great precision (<1 mm^3^) and novel acquisition protocols allow imaging of different tissue properties (in structural and functional modalities). This engineering technology has been combined with advances in analytical techniques (Bates *et al.*, 2003; Tyler *et al.*, 2005) resulting in increasingly sophisticated, detailed lesion–symptom mapping (though subject to significant challenges: cf. Mah *et al.*, 2014) and the evaluation of lesion-based prediction models.

Lesion mapping methodologies have been used widely in the stroke aphasia literature (e.g. Bates *et al.*, 2003; Schwartz *et al.*, 2009; Geva *et al.*, 2011; Magnusdottir *et al.*, 2013; Mirman *et al.*, 2015*a*; Yourganov *et al.*, 2016). While the precision of the structural imaging has been improved dramatically, there is an underlying issue that is rarely addressed: damage to a given area of the brain after stroke is not random but constrained by the neurovasculature. This means that there is neither full nor random sampling of the brain, which would be the ideal situation for lesion–symptom mapping. In addition, the non-random nature of stroke means that there is some degree of co-linearity between neighbouring voxels, which can bias or mislocalize lesion–symptom relationships (Inoue *et al.*, 2014; Mah *et al.*, 2014). Classical angiography studies identified the regions supplied by the arterial branches as well as their variations across individuals (Michotey *et al.*, 1974; Newton and Potts, 1974). For the cognitive and language deficits associated with middle cerebral artery (MCA) stroke, the most pertinent branches are probably those related to the subcortical regions (arising from M1), the insular (arising at M2) and the eleven cortical branches (arising from M1 and M4), including: (i) orbitofrontal, (ii) prefrontal, (iii) precentral, (iv) central, (v) anterior parietal, (vi) posterior parietal, (vii) angular, (viii) temporo-occipital, (ix) posterior temporal, (x) middle, temporal and (xi) anterior and polar temporal arteries. Accordingly, voxels that fall into each of these specific regions are likely to be damaged together and, in turn, there will be correlations between certain cortical regions due to the bifurcation/trifurcation of the MCA (e.g. occlusion of the superior branch will affect prefrontal and motor regions but not those of the temporal lobe).

The key aim of this study, therefore, was to use a data-driven approach to uncover the underlying patterns in the lesions of 70 chronic aphasic post-stroke cases. We achieved this aim by using principal component analysis (PCA) with varimax rotation to investigate the patients’ lesion structure. One previous study on coma used PCA on brain images to compare with a probabilistic frequency map (Singhal *et al.*, 2012). They identified six components in the patients’ data and concluded that the PCA methodology was better equipped to depict patterns of co-varying damage than probabilistic frequency maps. In addition, we tested whether the new parcellation could reduce the displacement error observed in single-voxel lesion–symptom mapping by replicating a pipeline developed in a recent study (Mah *et al.*, 2014). We compared the lesion-based parcellations and voxel-based results to another widely used anatomical parcellation, Brodmann areas (BAs), as a control.

Finally, we also conducted an initial exploratory analysis to explore how the data-driven lesion-based parcellations relate to the patients’ behavioural variations. In previous work, multiple research groups have used PCA with varimax rotation to unpack complex behavioural variations (Vandenbulcke *et al.*, 2005; Fucetola *et al.*, 2009; Wilson *et al.*, 2010; Kümmerer *et al.*, 2013; Butler *et al.*, 2014; Corbetta *et al.*, 2015; Mirman *et al.*, 2015*a*; Nair *et al.*, 2015; Siegel *et al.*, 2016; Halai *et al.*, 2017; Lacey *et al.*, 2017). The main advantage of applying varimax rotation is to improve interpretability by rotating the principal components such that assessments load highly on one component and minimally on others (Jolliffe, 2002). When applied to data collected from a large group of chronic, post-stroke aphasics across a set of 21 detailed language and cognitive assessments, we previously identified four factors that included phonological ability, semantic ability, executive demand and speech fluency (Butler *et al.*, 2014; Halai *et al.*, 2017). Similar data structures have been obtained in independent patient samples (Kümmerer *et al.*, 2013; Mirman *et al.*, 2015*a*, *b*; Alyahya *et al.*, 2018; Halai *et al.*, 2018). This study utilized the same deconstruction of the patients’ behavioural data and compared two different forms of exploratory lesion–symptom analyses: (i) the more traditional approach based on voxel analyses and (ii) an investigation of the potential relationship between the behavioural and lesion PCA structures.

## Materials and methods

### Participants

This study included 70 post-stroke patients with chronic aphasia (either ischaemic or haemorrhagic) with damage restricted to the left hemisphere (see Supplementary Table 1 for patients’ background information). Inclusion criterion for recruiting patients were: (i) monolingual native English speakers, (ii) normal or corrected-to-normal hearing and vision, (iii) right handed, (iv) one stroke, (v) at least 12 months’ post-stroke, (vi) no other known neurological conditions, (vii) no contradistinctions for MRI scanning and (viii) chronic aphasia of any type or severity. Informed consent was obtained from all participants under approval from the local ethics committee. Structural imaging data from a healthy age and education-matched control group (8 females, 11 males) were used to determine the lesion outline in the patients using an automated lesion identification procedure (Seghier *et al.*, 2008). We note that a subset of patients (*N* = 31) was used in the previous studies (Butler *et al.*, 2014; Halai *et al.*, 2017).

### Neuropsychological assessments

To test the participants’ speech, language and cognitive abilities, we utilized a detailed neuropsychological test battery, designed to assess input/output phonological processing, semantic processing and sentence comprehension, as well as general executive-cognitive function. The battery included a subset of tasks from the psycholinguistic assessments of language processing in aphasia battery (Kay *et al.*, 1992): (i) auditory discrimination using non-word minimal pairs, (ii) auditory discrimination using word minimal pairs, (iii) immediate repetition of non-words, (iv) immediate repetition of words, (v) delayed repetition of non-words and (vi) delayed repetition of words. Tasks from the 64-item Cambridge Semantic Battery (Bozeat *et al.*, 2000) were included: (vii) word-to-picture matching task (spoken version), (viii) word-to-picture matching task (written version), (ix) Camel and Cactus Test (picture) and (x) picture naming test. Other language tasks included (xi) the Boston naming test (Kaplan *et al.*, 1983), (xii) written 96-trial synonym judgement test (Jefferies *et al.*, 2009), (xiii) the spoken sentence comprehension task from the comprehensive aphasia test (CAT) (Swinburn *et al.*, 2005) and the ‘Cookie theft’ picture description task from the Boston Diagnostic Aphasia Examination (Goodglass and Kaplan, 1983). Specifically, patients’ responses in the ‘Cookie theft’ picture description task were recorded and transcribed. The (xiv) number of word tokens (T), (xv) type/token ratio, (xvi) mean length of utterance in morphemes and (xvii) words-per-minute were computed. Cognitively related tasks included (xviii) forward and (xix) backward digit span (Wechsler, 1987), (xx) the Brixton Spatial Rule Anticipation Task (Burgess and Shallice, 1997) and (xxi) Raven’s Coloured Progressive Matrices (Raven, 1962). All scores were converted into percentage based on the maximum score available; where no maximum was available, we used the maximum score in the group. The testing took place over a number of testing sessions, where the pace was determined by the participant and each session lasted 1 h and 30 min. The testing pipeline was as follows: (i) initial consent and start testing battery, (ii) MRI scan and (iii) continue behavioural testing to completion, where all testing was completed within 2 months of starting the testing.

### Acquisition of neuroimaging data

High-resolution structural T_1_-weighted MRI scans were acquired on a 3.0-T Philips Achieva scanner (Philips Healthcare, Best, The Netherlands) using an eight-element SENSE head coil. A T_1_-weighted inversion recovery sequence with three-dimensional acquisition was employed, with the following parameters: repetition time = 9.0 ms, echo time = 3.93 ms, flip angle = 8°, 150 contiguous slices, slice thickness = 1 mm, acquired voxel size 1.0 × 1.0 × 1.0 mm^3^, matrix size 256 × 256, FOV = 256 mm, inversion time = 1150 ms, SENSE acceleration factor 2.5 and total scan acquisition time = 575 s.

### Preprocessing neuroimaging data

**Figure 1:**
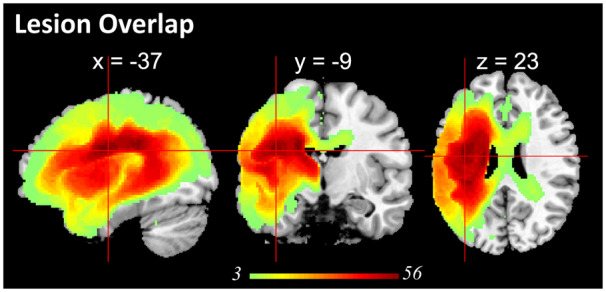
**Lesion profile of the patient group.** Subcortical regions and white matter, e.g. putamen, corpus callosum, thalamus, inferior occipito-frontal fasciculus, caudate, internal capsule, insular, had the largest probability of being damaged.

High-resolution structural scans were pre-processed with the same procedure as our previous studies (Butler *et al.*, 2014; Halai *et al.*, 2017), with one additional first step. We extracted the brain space for all T_1_ images using a brain extraction tool optimized for patient brains (Lutkenhoff *et al.*, 2014). This step was performed to avoid edge artefacts during smoothing where non-brain tissue with high intensity would be smoothed into brain tissue. We obtained a mask in native space and used the following procedure to normalize the whole T_1_ image (not brain extracted) and use the transformation matrix to warp the mask into Montreal Neurological Institute space. The analysis used Statistical Parametric Mapping software (SPM8: Wellcome Trust Centre for Neuroimaging, http://www.fil.ion.ucl.ac.uk/spm/) and a modified unified segmentation–normalization procedure (Seghier *et al.*, 2008). After normalizing individual lesioned brain images into standard Montreal Neurological Institute space, images were smoothed with an 8-mm full-width-half-maximum Gaussian kernel. Lesions were automatically identified for each patient by comparing the structural image with the age- and education-matched control group, using an outlier detection algorithm to identify ‘abnormal’ voxels (Seghier *et al.*, 2008). All parameters were kept at default except the lesion definition ‘U-threshold’, which was set to 0.5 after comparing the results obtained from a sample of patients to what would be nominated as lesioned tissue by an expert neurologist. The method produces two types of outputs: an abnormality likelihood map and a binary lesion map. In the abnormality likelihood map, the signal intensity for any given voxel is compared to the distribution of signal in the same voxel from a control group where a higher value indicates that the voxel is more likely to be abnormal (scaled 0–1), which will include vascular and subsequent atrophic changes. The binary lesion maps where defined using the ‘U-threshold’ and a cluster extent of 100 voxels (i.e. in our case the voxels had to have at least 50% likelihood of being abnormal and form clusters ˃100 voxels). The abnormality likelihood maps were used in the PCA, and the binary lesion maps were only used to show the distribution of lesions within our dataset (Fig. 1).

### Preliminary analyses of voxel-wise lesion to behavioural variations

We performed a PCA using SPSS 22. The input data consisted of 21 behavioural scores for 70 patients. Factors were extracted based on the correlation matrix and components with eigenvalues ˃1.0 were varimax rotated. Individual patient scores for each factor were obtained using the regression method. These factor scores were then entered into the same model (as they are orthogonal) and correlated to the lesion profile based on T_1_-weighted images at the voxel-wise level (as done in Butler *et al.*, 2014; Mirman *et al.*, 2015*b*; Halai *et al.*, 2017; Lacey *et al.*, 2017). Clusters were significant at AlphaSim corrected *P *<* *0.01 with a voxel threshold of *P *<* *0.001.

### Preliminary analyses of the relationships between PCA-based lesion components and behavioural variations

We conducted the PCA of the lesion data in MATLAB (R2012a) and used the minimum description length criteria and Kolmogorov information criterion (Akaike, 1974; Rissanen, 1983) (from GIFT MATLAB software, http://icatb.sourceforge.net/) to estimate the number of components within the data that explained the most variance with a penalty for increasing the number of components. We used the ‘princomp’ function to extract the components and then applied a varimax rotation to help with the interpretation of the resulting components (using the ‘rotatefactors’ function). We projected the PCA factor scores back into brain space and converted the data into *z*-scores and used |*z|* > 3.29 (*P *<* *0.001 two-tail) as the cut-off value. Clusters ˃500 voxels (volume: 4 cm^3^) were reported using the Harvard–Oxford (cortical and subcortical) and natbrainlab (white matter) atlases. Having obtained the structure of the underlying lesions, we explored which lesion component(s) were related to the behavioural variations. First, we conducted Pearson correlation analysis between patients’ 20 lesion component scores and 4 behaviour component scores (Bonferroni corrected at *P *<* *0.05). To test which of the lesion components were the most important predictors, we built a stepwise regression model where the lesion component scores were used to predict the behavioural component scores. The lesion component(s) that significantly predicted behavioural factors were then mapped onto the brain and converted into *z*-scores [|*z|* > 3.29 (*P *<* *0.001 two-tail) as the cut-off value]. Clusters ˃200 voxels (volume: 1.6 cm^3^) were reported using the Harvard–Oxford (cortical and subcortical regions) and natbrainlab (white matter) atlases.

### Assessing spatial error using different simulations and methods

We conducted two analyses to help determine how well the lesion territory parcellation fared compared to a single-voxel or Brodmann atlas model. First, we determined the displacement error induced by different lesion models by changing the ground truth (at single-voxel, BA and lesion territory parcellation level). Similar analyses in the literature have used different parcellations, which contain brain regions of differing sizes (e.g. Inoue *et al.*, 2014; Mah *et al.*, 2014; Pustina *et al.*, 2018). We hypothesized that the parcellation that produces the smallest displacement error will be the one that most closely reflects the underlying nature of stroke-induced brain damage. In the first instance, we simulated the ground truth at the voxel level, meaning that one single voxel is critical to certain behaviour. For this voxel, we split our sample into two groups based on whether the voxel was damaged and performed mass-univariate analyses to search for other voxels whose damage was associated to the ground truth. Fisher’s exact test was used to determine statistical inference, where the resultant map was thresholded at *P *<* *0.01, false discovery rate (FDR) corrected for multiple comparisons. We calculated the centre of mass of the significant cluster and determined the Euclidean distance between this point and ground truth voxel. This process was repeated for 57 632 voxels, for which more than three patients had damage. Next, we simulated the ground truth using BAs, where for each area the sample was split into intact/damaged based on at least 20% of the area being damaged and a case with damage had a 90% chance of exhibiting a behavioural deficit (Mah *et al.*, 2014). The same lesion mapping method was used in the single-voxel pipeline (Fisher’s exact test, *P* < 0.01, FDR corrected for multiple comparisons), and the resulting cluster was used to calculate the displacement error based on the centre of gravity. This process was repeated for 29 areas, which had at least three patients with damage. Finally, the same process was repeated using the lesion principal components (the vascular territories). A Welch *t*-test was used to statistically compare the displacement values across the three lesion models.

In the second set of analyses, we compared the spatial errors based on models that used mass univariate analysis or multivariate methods [we chose sparse canonical correlation for neuroimaging (SCCAN); Pustina *et al.*, 2018] and compared the spatial error obtained using the PCA components based on stepwise regression and separately using correlations. Due to computational constraints for calculating these models at the single-voxel level, we focused on the BAs used in Mah *et al.* (2014), which included BA 37 and 38 separately, 37 plus 38 combined and 39 plus 44 combined. First, we simulated ground truth brain–behaviour relationships using BAs in the same way as described in Mah *et al.* (2014). We calculated two SCCAN results: (i) using a fixed sparseness value used previously (0.045) and (ii) the optimal sparseness using cross-validation between 0.005 and 0.9 values (Pustina *et al.*, 2018). The mass univariate analysis and PCA plus regression/correlation were conducted in the same way as described above. Since we tested distributed regions (e.g. lesion in BA 37 or BA 38 related to behaviour deficits) and the centre of mass is no longer meaningful, we used Dice similarity between the ground truth and significant regions found with different methods as a measure of spatial error.

### Data availability

The conditions of our ethical approval do not permit the public archiving of anonymized study data. The anonymized data necessary for reproducing the results in this article can be requested from the corresponding author.

## Results

### Neuropsychological and lesion distribution

A summary of the patients’ scores is provided in Supplementary Table 1. The sample contained a range of aphasic performance from global/severe to well-recovered cases. The patients’ lesion overlap map is provided in Fig. 1 and primarily covered the left hemisphere area supplied by the MCA (Phan *et al.*, 2005). The automated lesion identification procedure also identifies vascular and subsequent atrophic changes. The maximum number of participants who had a lesion in any one voxel was 56 (Montreal Neurological Institute coordinates −38, −9, 24; anatomy of peak: anterior segment of arcuate fasciculus).

### Behaviour factors

The factor analysis on the behavioural data revealed four orthogonal dimensions (see Table 1), which replicates findings from previous studies that had less than half the number of participants (Butler *et al.*, 2014; Halai *et al.*, 2017). The degree to which each test loaded on the factors allows us to interpret the cognitive meaning of each factor. The first factor loaded highest with repetition but also with naming and digit span (and weakly with spoken sentence comprehension), suggesting that this factor relates to phonological ability. The second factor loaded highest with picture matching but also with synonym judgement, Camel and Cactus Test and type/token ratio, suggesting that this factor relates semantic processing. The third factor loaded highest with minimal pairs, Raven’s Coloured Progressive Matrices and the Brixton spatial anticipation test, suggesting that this factor relates to executive-related problem-solving or decision-making processes. The fourth factor loaded highest with the number of speech tokens produced but also with words per minute and mean length of utterances, suggesting that this factor relates to the quantity of speech produced.

**Table 1:** Loadings of behavioural assessments on factors extracted from the rotated PCA

Tasks	Component			
	Phonology	Semantics	Executive	Fluency
Delayed repetition—words	**0.888**	0.221	0.183	0.193
Delayed repetition—non-words	**0.883**	0.027	0.237	0.148
Immediate repetition—non-words	**0.881**	0.061	0.231	0.143
Immediate repetition—words	**0.858**	0.211	0.125	0.170
Boston naming test	**0.823**	0.381	0.077	0.121
64-Item naming	**0.813**	0.431	0.154	0.117
Forward digit span	**0.746**	0.233	0.188	0.073
Backward digit span	**0.595**	0.207	0.234	0.359
CAT spoken sentence comprehension	**0.521**	0.455	0.441	0.163
Spoken word to picture matching	0.236	**0.801**	0.267	0.145
Type/token ratio	0.362	**0.718**	−0.075	−0.092
Written word to picture matching	0.182	**0.713**	**0.504**	0.155
Camel and Cactus Test: pictures	0.092	**0.688**	0.484	0.288
96 synonym judgement	0.381	**0.658**	0.315	0.359
Minimal pairs—non-words	0.353	0.058	**0.814**	−0.014
Raven’s Coloured Progressive Matrices	0.048	0.274	**0.735**	0.156
Minimal pairs—words	0.419	0.168	**0.705**	0.132
Brixton spatial anticipation test	0.132	0.178	**0.698**	0.231
Token	0.010	0.034	0.207	**0.885**
Mean length of utterance in morphemes	0.314	0.252	0.137	**0.831**
Words per minute	0.314	0.096	0.080	**0.768**

Factor loadings >0.5 are given in bold. CAT = comprehensive aphasia test.

### Lesion principal components

Before reporting the results, it should be noted that the sign (positive or negative) of the coefficients is arbitrary, since variance does not depend on sign; however, the order of the components is important, with the first component accounting for the most variance and the last component the least. The minimum description length criteria (Akaike, 1974; Rissanen, 1983) suggested that there were 20 components within the current dataset. We also used the Kolmogorov information criterion, which generated an estimate of 32 components within the dataset. For clarity, we only show the results for the 20 component model in the main manuscript but have provided the results of the 32 component model in the Supplementary Materials. In brief, the cluster pattern remained the same but several big clusters breakdown into smaller, more scattered clusters, which are much less interpretable than the 20-component solution (see Supplementary Fig. 2 for all 32 components). Minimal description length is frequently used in ICA studies for the estimation of dimensions and has also been used with PCA. As noted above, this method suggested 20 components for our patient group. We performed a follow-up permutation analysis to determine how many components would be estimated by randomly selecting 5, 10, …, 65 and 70 patients from the whole patient group. The permutation test was repeated 100 times for each sample size. The result is provided in Supplementary Fig. 3. This analysis showed that when using smaller sample sizes, the number of estimated components varies widely but the number stabilizes for larger patient samples. The median component number for a sample size of *N* = 40 was 17, while for *N* = 60 it was 19. For a sample size *N* = 65 and 70, the median value was 20 components. This result suggests that there are likely to be 20 components and, accordingly, this is the value we used in the study.

**Figure 2:**
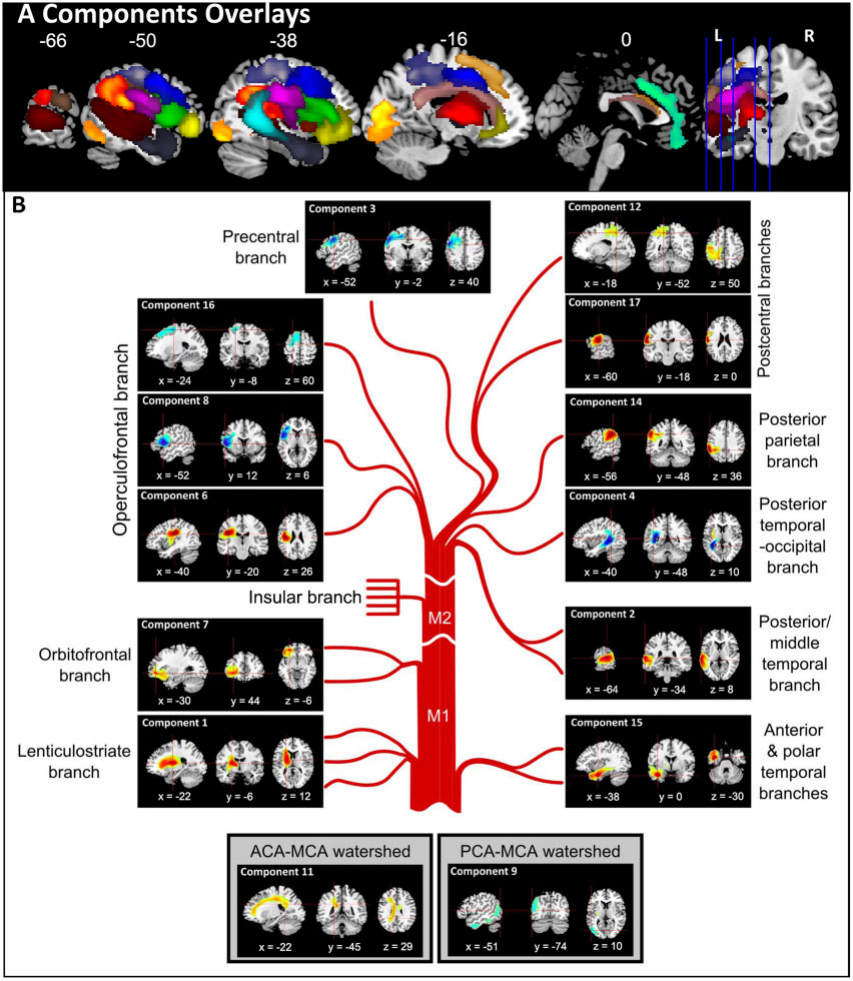
**Principal component analysis of abnormality likelihood maps.** (**A**) Components overlays. Components 1–8 and 11–17 are shown on the same brain template. (**B**) Individual components of MCA illustrated in detail—note the brief labels refer to visually-matched MCA components but may well also reflect other important vascular individual differences such as alternative branching and variable watershed regions (see main text for descriptions).

The components could be grouped based on location within the fronto-parietal lobes or temporal lobe. Most components belonged to the MCA territory and were located in the fronto-parietal lobes, where Component 1 was subcortical, Components 3, 6, 7, 8, 12, 16, and 17 were frontal and Component 14 was parietal. There were three components in the temporal lobe (Components 2, 4 and 15). Two or three components were located along the watershed between territories (Components 9 and 11 and possibly 16). Component 13 fell within the medial frontal region and was likely to belong to the anterior cerebral artery territory and Component 9 in the occipital lobe was likely to belong to the posterior cerebral artery territory. The four other components were either scattered or included regions in the right hemisphere and thus hard to interpret (Components 10, 18, 19 and 20; see Supplementary Fig. 1 for all 20 components). A visual illustration of the components is shown in Fig. 2 where we arranged the lesion components based on the vasculature (a detailed description of the components’ locations is given in Supplementary Table 2). In the following paragraphs, we describe the location of each cluster in relation to anatomy using the Harvard–Oxford (grey matter) and natbrainlab (white matter) atlases. Given that the seminal angiography studies of the MCA sub-branches were conducted before three-dimensional brain mapping had been developed, we visually compared the PCA-derived clusters and the hand-drawn maps from Michotey *et al.* (1974). It is important to note that the PCA will also detect other sources of consistent individual differences in vascular supply and damage that is related to individual variations in vascular branching, patterns of vascular collateralization, and so on. Perhaps most importantly for the study it is worth noting that the key aim of this approach is to investigate the possibility of moving away from voxel-based analysis to one that embraces the collinearities in voxel damage. In this context, it is more powerful that the PCA outcome is a reflection of all sources of variance found in the patient sample in addition to the typical vascular branching itself.

The first component was located in subcortical territories that mainly occupy the putamen and thalamus, and underlying white matter, e.g. the cortical spinal and the inferior occipito-frontal fasciculus. Thus, it corresponded to the lenticulostriate branch, originating from the M1 segment of the MCA. The current data set did not discover a pure insular branch, which originates from the M2 segment of the MCA. Components 8 and 6 corresponded to the operculofrontal branch, originating from the M3 segment. Component 8 was located at the frontal operculum cortex, extending to inferior frontal gyrus (pars opercularis) and precentral gryus. Component 6 was located at the central opercular cortex, extending to insular and parietal operculum cortex. Components 7, 3, 17 and 12 corresponded to the orbitofrontal, precentral, central and postcentral branches, respectively. These four are the territories of the frontal cortical branches, originating from the M4 segment. Component 7 came after the subcortical branch, located at the orbital frontal cortex, extending to frontal pole and inferior frontal gyrus (pars triangularis). Following it was Component 3, located at the middle frontal gyrus, extending to the precentral gyrus and inferior frontal gyrus. Component 17 was lateral to Component 3, lying on the central sulcus, occupying both precentral and postcentral gyri. Component 12 was posterior to Component 3 and was located at the postcentral gyrus, but occupying a large section of the superior parietal lobule. Component 16 was located at the middle and superior frontal gryus and was superior to the other frontal components. It is possible that this either reflected the most dorsal frontal branch or belonged to the watershed with the anterior cerebral artery. Component 14 was completely within the parietal lobe, including supramarginal gyrus and angular gyrus, and was linked to the posterior parietal branch.

Moving inferiorly into the temporal lobe territories, we found Component 2, which was in the lateral temporal lobe, extending from the anterior superior and middle temporal gyrus to the angular gyrus and supramarginal gyrus. As the peak was posterior, we suggest that this reflects the posterior temporal branch. The medial temporal lobe structures made up Component 4, including the inferior longitudinal fasciculus and posterior segment of the arcuate fasciculus, therefore was likely to reflect the posterior temporal–occipital branch. We note that this component covered the ventricle and possibly reflected atrophic changes. The anterior inferior temporal lobe made up of Component 15, including the anterior portion of the inferior longitudinal fasciculus. The one component in the occipital lobe (Component 5) was located over the inferior division of lateral occipital cortex, occipital fusiform gyrus and occipital pole and, therefore, was more likely to belong to the posterior cerebral artery.

We labelled Components 9 and 11 as watershed components as they overlapped with the territory between cerebral arteries (Michotey *et al.*, 1974). Component 9 overlapped with the watershed territory between MCA and posterior cerebral artery, occupying the lateral occipital cortex down to the middle and inferior temporal gyrus. Although both Components 5 and 9 are located within occipital regions, we do not believe that they reflect similar underlying territories as Component 5 is more focal compared to Component 9. Component 11 overlapped with the watershed territory between MCA and anterior cerebral artery, occupying white matters including the corpus callosum and cingulum. This component also extends into the right ventricle and therefore might also capture atrophy related to the effects of long-standing vascular load. Component 10 may partially reflect the cortical portion of the watershed between anterior cerebral artery and MCA, as it covers a range of regions along the frontal pole and middle frontal gyrus but the cluster in the right hemisphere makes it difficult to interpret.

### Voxel-wise neural correlates of the behaviour factors

**Figure 3:**
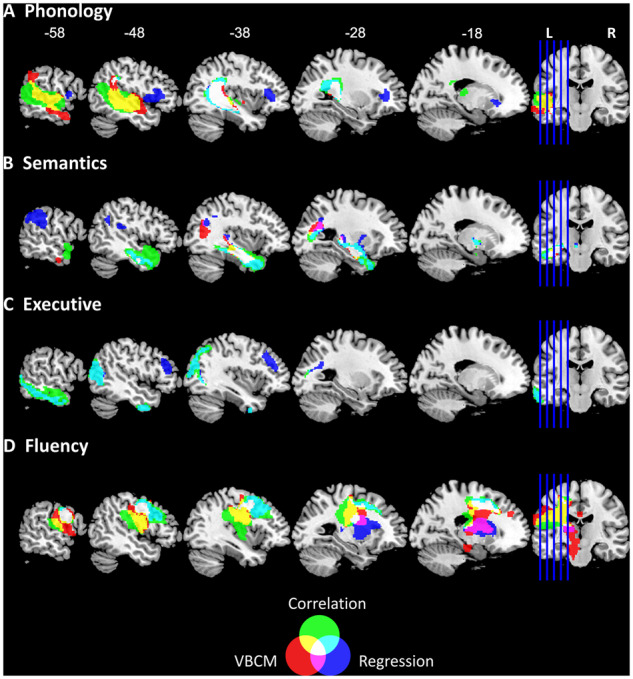
**Lesion–symptom mapping using voxel-based correlational methodology, lesion component correlation and regression.** Red colour denotes results of the voxel based correlational analysis with behaviour components (AlphaSim corrected *P *<* *0.01 with voxel *P* < 0.001). Blue colour denotes regression analysis using lesion components to predict behaviour factor scores (all regression models significant at *P *<* *0.001). Green colour denotes the Pearson correlation between lesion components and behaviour components (all correlation significant at *P *<* *0.0006). The overlapping regions between the analyses are colour-coded according to the legend (Venn diagram).

The neural correlates identified using voxel-based correlational methodology (VBCM) for phonology, semantics and fluency are shown in Fig. 3 (red regions) and described in Supplementary Table 3 (AlphaSim corrected *P *<* *0.01 with voxel *P *<* *0.001). There were no significant clusters for the executive factor at this statistical threshold. The cluster related to phonology was found in the dorsal auditory language pathway, including middle and superior temporal gyrus, extending posterior to supramarginal gyrus and medial whiter matter substrates, e.g. inferior longitudinal fasciculus and arcuate posterior segment (5641 voxels; peak coordinates: −64, −18, −12; peak *r *=* *0.55, *P *<* *0.001). Semantic ability was related to two clusters. The first one covered the lateral and medial anterior temporal lobe and extending to medial temporal lobe inferior longitudinal fasciculus (1175 voxels; peak coordinates: −36, −12, −18; peak *r *=* *0.54, *P *<* *0.001). The second cluster was at the posterior end of the inferior longitudinal fasciculus and extended into the superior division of lateral occipital cortex (509 voxels; peak coordinates: −34, −66, 18; peak *r *=* *0.46, *P *<* *0.001). Finally, the fluency factor correlated with a large cluster in the frontal lobe, covering the precentral and postcentral gyrus, thalamus, central opercular cortex and also white matter including the frontal aslant tract, cortico-spinal, corpus callosum and cingulum (9479 voxels; peak coordinates: −60, −6, 20; peak *r *=* *0.57, *P *<* *0.001).

### Relating lesion component scores to the behavioural factor scores

The Pearson correlation analysis (Fig. 3 green regions) between lesion components and behavioural components showed that the correlated components overlapped with the VBCM results. We found that Components 2 (*r* = −0.409) and 4 (*r *=* *0.419) correlated with phonology components score, Component 15 (*r* = −0.495) correlated with semantics, Component 9 (*r *=* *0.477) correlated with executive function and, lastly, Components 3 (*r *=* *0.436) and 6 (*r* = −0.407) correlated with fluency (all *P *<* *0.0006).

We used stepwise regression to determine if the lesion components can be used to predict behavioural factors. The results are shown in Fig. 3 (blue clusters) and subsequent details for the clusters are shown in Supplementary Table 4. We found a significant model for predicting phonological ability (*F*(2,67) = 10.288, *P *<* *0.001), which included Component 4 (beta = 0.429) and Component 8 (beta = 0.244), with an adjusted *r* square of 0.212. We mapped the *z*-transformed coefficients back into brain space and found two significant clusters. The largest cluster was located over the left inferior longitudinal fasciculus and the posterior segment of the arcuate fasciculus, while the second cluster was located in the left pars opercularis and frontal operculum cortex. A significant model predicted semantic ability [*F*(3,66) = 11.640, *P *<* *0.001], which included Component 15 (beta = −0.418), Component 14 (beta = −0.336) and Component 12 (beta = 0.256), with an adjusted *r* square of 0.316. We found three significant clusters on the brain, of which the largest cluster was located within the left temporal lobe and the inferior longitudinal fasciculus. The second cluster was identified in the left supramarginal gyrus, and the third cluster was in the left optic radiations. We observed a significant model for executive ability [*F*(2,67) = 14.152, *P *<* *0.001], which included Component 9 (beta = 0.443) and Component 10 (beta = −0.266), with an adjusted *r* square of 0.276. Neural regions related to the executive factor were located in the watershed regions. The largest cluster overlapped with inferior temporal lobe running posterior to the occipital lobe, while the second cluster was located within the left middle frontal gyrus, the frontal pole and the inferior frontal gyrus. Finally, a significant model was identified for speech fluency ability [*F*(2,67) = 11.665, *P *<* *0.001], which included Component 3 (beta = 0.352) and Component 1 (beta = −0.274), with an adjusted *r* square of 0.236. The neural results revealed two significant clusters, the largest of which was located in the left middle frontal gyrus and precentral gyrus. The smaller cluster was found in subcortical regions such as the cortico-spinal tract, putamen and thalamus.

### Assessing spatial error using different simulations and methods

We conducted two analyses to test how useful the PCA territories were, compared with a single-voxel model and another whole brain atlas (Brodmann map). In the first instance, we simulated different ‘ground truths’ using a single voxel, BA or vascular territories. We found that the mean displacement error was largest for the Brodmann parcellation (22.46 mm, standard deviation = 16.28 mm), followed by the single-voxel model (16.34 mm, standard deviation = 6.87 mm) and then the vascular territories model (13.85 mm, standard deviation = 4.65 mm). The displacement of the vascular territories was significantly smaller than the single-voxel simulation (Welch’s *t*-test, *t *=* *2.14, *P *=* *0.0493, two-tailed) and the Brodmann parcellation simulation [Welch’s *t-*test, *t *=* *2.66, *P *=* *0.0117, two-tailed; see Supplementary Fig. 4 for a violin plot of the displacement error for each model and a three-dimensional brain projected figure showing which areas of the brain have high/low displacement; for reference, we also show the probability of damage following an MCA stroke by Phan *et al.* (2005)].

In the second set of analyses, we compared the spatial error for three modelling methods (mass univariate, SCCAN or PCA regression) when using a specific set of ground truth ROIs based on the Broadmann map [BA 37 and 38 alone, BA 37 plus 38 and BA 39 plus 44; replicating the models reported by Mah *et al.* (2014)]. This result did not reveal a standout method based on the Dice values (see Supplementary Fig. 5). We found that the SCCAN results were very similar to the mass univariate results when applying a conservative threshold, or when capping the number of voxels to match the SCCAN results. The PCA regression and correlation models always identified a cluster that overlapped with the ground truth ROI but, given that the vascular components are much larger (regions rather than individual voxels), the end result always extended beyond the ground truth. Despite this disadvantage, the PCA regression and correlation method performed better than SCCAN in one out of the four simulations (for BA 37). Finally, it is worth highlighting that the significant regions identified by all three methods did not neatly conform to the BAs; in the multiple region simulation, all methods identified a large region extending outside of the ground truth.

## Discussion

Modern neuroimaging now allows sophisticated *in vivo* lesion mapping. Many previous studies using lesion–symptom mapping have not taken into account the non-random distribution of brain damage after stroke, which is constrained by the vasculature. In the present study, we showed that PCA of the lesions in post-stroke aphasia can reveal the underlying components of damage across the MCA territory, which are striking similarity to the vascular structure and forms of vascular individual variation identified by seminal angiography studies (Michotey *et al.*, 1974). This parcellation produced less displacement during lesion–symptom mapping inference compared to a standard single-voxel model and an anatomical parcellation, which suggests that taking into account the vascular territories might improve our understanding of the locus of behavioural deficits in stroke patients. In a secondary stage, we undertook a preliminary exploration of how these lesion components might relate to the patients’ language and cognitive variations.

### Comparing the lesion principal components with angiography

It is striking that the majority of components, identified by varimax-rotated PCA of the neural data, were clusters that reflect many of the cortical and subcortical MCA territories (see Fig. 2) identified in classical angiography studies as well as other forms of vascular individual differences (Michotey *et al.*, 1974). We should note that we labelled the PCA clusters by visual comparison with the descriptions provided in these previous seminal studies. These classical studies were conducted long before formal methods had been developed for three-dimensional brain mapping that would allow definitive direct comparisons between the vascular supply of the MCA sub-branches and PCA-derived clusters. As expected, the lesion components showed an anterior–posterior separation covering frontal and temporoparietal regions. Previous angiography studies identified four branches in the frontal lobe (orbitofrontal, prefrontal, precentral and central), which were reflected in six PCA components across both lateral and medial frontal regions. We found direct correspondence for the two parietal branches (Components 12 and 14). Component 12 covered superior parietal lobule and extends into postcentral gyrus, while Component 14 was situated over the angular and supramarginal gyri. We also noted a high degree of overlap between the PCA components within the temporal lobe and classical angiography studies. The PCA identified three components along the caudal–rostral axis (Components 2, 4 and 15). One possible reason for detecting larger temporal principal components in the lesion maps than expected by the angiography studies may be due to the fact that our patient distribution is such that we have relatively fewer cases with damage to the temporal lobe than frontal lobe. This, in turn, may reflect the fact that the middle and ventral parts of the temporal lobe are less likely to be damaged following an MCA infarct: Phan *et al.* (2005) identified that the likelihood of damage dropped to 10–15% in the middle temporal gyrus and <5% from the inferior temporal gyrus and below. In contrast, the probability of frontal-insular damage is ˃30%. It is also possible that the hierarchical branching and diameter of the sub-branches is such that certain combinations are likely to be jointly occluded (e.g. the polar and anterior temporal branches). The lesion PCA also identified two other major features of the vascular supply. First, as well as the MCA cortical territories, we also obtained the lenticulostriate branch (Component 1) as well as the watershed territories between middle and posterior cerebral artery (Component 9) and middle and anterior cerebral artery (Component 11), which are known to show individual variability (Michotey *et al.*, 1974), and thus these regions may be less likely to emerge in lesion–symptom mapping analyses. Second, the analyses also identify more widespread atrophic changes, which may well reflect the effects of long-term vascular load.

We note that, inescapably, the spatial maps of principal components depend on the number of the components extracted. When a different optimization criterion was applied, a larger estimated number of components were derived yet the maps relating to these components were much harder to interpret and did not relate obviously to the known cortical vascular supplies (see Supplementary Fig. 2). The number of components was also much lower than the number of regions found in many brain parcellation approaches based on cytoarchitecture, function or connectivity. We suspect that this variation reflects differences in the inherent granularity of the raw data in each case. Our estimate of 20 vascular components aligns with the known number of cortical vascular branches of the MCA (*N* = 12) plus individual differences in vascular supply. In contrast, there are numerous variations in cytoarchitecture or cortical functions and, thus, it seems inevitable that there will be many more regions in those resultant parcellations of the brain. Future studies that have access to much larger lesion databases can assess that the spatial clusters identified in this study are efficient in capturing the post-stroke damage, by evaluating predictive modelling of out-of-sample data.

### Impact of vascular territory parcellations in lesion–symptom mapping

We provided two sets of results (i.e. manipulating the ground truth and manipulating the inference method) to help understand the effect on lesion–symptom mapping. Our results showed that, when we altered the ground truth, the vascular territories parcellation produced smaller displacement to the simulated ground truth compared to a single-voxel or Brodmann map model. The displacement values for the single-voxel model and BAs derived in the current study are very similar to recent studies (i.e. Mah *et al.*, 2014; Sperber and Karnath, 2017). By projecting the displacement values to the brain, we identified that the regions with greatest displacement were typically located on the edge of the MCA territory between the other two major arteries (Michotey *et al.*, 1974). We offer two possible explanations for the increased displacement in these regions: (i) the number of lesions in these territories is lower and thus the estimations are noisier or (ii) the boundaries between watershed territories are inherently variable across individuals, especially as dual supplied regions (such as the anterior temporal lobe and orbitofrontal cortex) would be more robust. In Supplementary Fig. 4C, we illustrate that the areas with the largest displacement are less likely to be damaged in an MCA stroke population (Phan *et al.*, 2005). This suggests that we may need to either include a very large population of patients to capture the <10% probability of damage to these regions sufficiently and/or include cases with strokes related to the other major arteries in conjunction with the MCA.

In the second set of analyses, we did not observe clear differences between the mass univariate or multivariate methods. This might appear contradictory to recent reports [e.g. support vector machine classifier in Mah *et al.* (2014) and SCCAN in Pustina *et al.* (2018)]; however, after close inspection of the results in the literature, there is a possibility that multivariate methods may not always be ‘better’ than univariate approaches. For example, a recent study found that lesion–symptom mapping results using mass univariate or multivariate (support vector) approaches were strikingly similar (Schumacher *et al.*, 2019). Furthermore, Sperber *et al.* (2018) reported that multivariate support vector methods are susceptible to spatial error to a similar extent as mass univariate analyses. Therefore, to date, it seems that it is unclear if multivariate methods outperform mass univariate analyses, and under which conditions they might differ. This is clearly an important topic for the field and more work is required to help improve our current brain-behavioural mapping methods.

### Comparing the neural correlates of behaviour at the voxel and lesion territory level

By the way of a preliminary exploration, we compared voxel-based analyses (VBCM) with the results from the lesion component to behavioural mapping. The results for phonological ability showed overlap between both methods in the posterior temporal region including the underlying posterior segment of the arcuate fasciculus, which has been shown to be involved in the dorsal auditory language pathway (Hickok and Poeppel, 2004; Catani *et al.*, 2005; Parker *et al.*, 2005; Hickok and Poeppel, 2007; Saur *et al.*, 2008; Ueno *et al.*, 2011). This region has also been implicated using other methodologies, e.g. diffusion-weighted imaging and tractography (Glasser and Rilling, 2008; Park *et al.*, 2011), intraoperative subcortical electrical stimulation (Duffau *et al.*, 2008; Leclercq *et al.*, 2010), neuroanatomically constrained computational models (Ueno *et al.*, 2011) and VLSM analysis (Bates *et al.*, 2003; Fridriksson *et al.*, 2011). There were some differences between the two methods. The VBCM analysis revealed correlations with a larger region including the lateral superior temporal gyrus and middle temporal gyrus, which is associated with the ventral language pathway (Hickok and Poeppel, 2004, 2007; Price, 2010). Although there is a lesion component (No. 4) that overlaps with this extended area, it did not appear in the regression analysis as the posterior temporal component (No. 2) explained a greater amount of variance and, thus, Component 4 did not enter the regression analysis. Consistent with this conclusion, the simple correlation analysis identified a larger temporal lobe region as correlated with phonological ability (Component 2 in addition to Component 4), which overlapped considerably with the area from the voxel-based analysis (see yellow-marked area in Fig. 3A). The lesion-component regression analysis also detected a second critical region for phonological abilities within the inferior frontal region. This follows from the fact that, having accounted for the variance along the temporal lobe, the regression analysis was able to identify additional independent variance associated with the inferior frontal component, which was not found in the VBCM or the simple correlations. Indeed, the inferior frontal lobe has been repeatedly linked with phonological processing in previous studies and has been associated with mapping sound structure to production and/or executive-attention mechanisms in selecting the correct sound mapping (Vigneau *et al.*, 2006; Rauschecker and Scott, 2009; Price, 2010).

VBCM and the behaviour-lesion components regression both revealed clusters in the white matter of anterior temporal lobe when mapping semantic ability. This finding converges with evidence that the anterior temporal lobe is important for successful semantic representation (Lambon Ralph *et al.*, 2017). The anterior temporal lobe has been suggested to act as a transmodal semantic hub, as revealed by multiple convergent methodologies, e.g. semantic dementia (Patterson *et al.*, 2007; Acosta-Cabronero *et al.*, 2011), repetitive transcranial magnetic stimulation (Pobric *et al.*, 2007), functional MRI (fMRI) (Visser *et al.*, 2010; Peelen and Caramazza, 2012; Coutanche and Thompson-Schill, 2015) and resting-state fMRI (Zhao *et al.*, 2017). Studies investigating the white matter connectivity, associated with the anterior temporal lobe have revealed that the uncinate fasciculus, inferior longitudinal fasciculus and inferior fronto-occipital fasciculus are related to semantic performance (Mandonnet *et al.*, 2007; Acosta-Cabronero *et al.*, 2011; Han *et al.*, 2013). The regression analysis was able to detect an extra region located within supramarginal and angular gyrus. These ventral parietal regions have been suggested to be related to semantic processing (Binder *et al.*, 2009; Hartwigsen *et al.*, 2015). However, there is growing recent evidence that the role of the angular gyrus might not be selective for semantic processing (Humphreys *et al.*, 2015) but rather may serve numerous cognitive domains (Humphreys and Lambon Ralph, 2015), including episodic memory (Wagner *et al.*, 2005; Rugg and Vilberg, 2013; Humphreys and Lambon Ralph, 2015).

Speech fluency is usually the first domain on which aphasia patients are categorized (Goodglass and Kaplan, 1983). In our previous behavioural work, we found that the quantity of speech produced can be separated from phonological and semantic abilities (Halai *et al.*, 2017). Classically, the inferior frontal gyrus (termed Broca’s area) had been associated with poor speech production (Broca, 1861); however, this has now been refined to the insula, precentral gyrus and subcortical structures such as the caudate, putamen and thalamus (Blank *et al.*, 2002; Bates *et al.*, 2003; Basilakos *et al.*, 2014; Halai *et al.*, 2017). The current study found overlap between the two lesion mapping methodologies, both identifying the precentral gyrus, middle frontal gyrus and thalamus. VBCM also found a wider region over the frontal lobe that extended laterally into inferior frontal gyrus as well as medially down the cortico-spinal tract. As with phonological ability, the broader region identified by VBCM was also found in the simple correlations (see yellow-marked region in Fig. 3D)—again reflecting the fact that in stepwise regression only the components that capture the unique sources of variance are included, while any components that have similar but slightly weaker explanatory power are disregarded.

Finally, VBCM analysis failed to identify any neural correlates for the executive factor (as also found in previous studies, unless low statistical thresholds are applied) (Butler *et al.*, 2014; Halai *et al.*, 2017; Lacey *et al.*, 2017). In contrast, the regression analysis identified regions at the edge of the MCA territory that predicted executive abilities. These identified regions align closely with the proposed fronto-parietal multi-demand network for executive function (e.g. Duncan, 2010). Furthermore, studies that extract network measures from resting-state fMRI and DTI have found a large-scale network of regions across the frontal, parietal and temporal lobe related to executive functions (Li *et al.*, 2009; van den Heuvel *et al.*, 2009). This distributed nature converges with the results observed in our analysis, whereby areas across all lobes are found to be predictive of better executive performance.

## Conclusion

Studies of cognitive and language impairments in stroke patients have provided valuable insights into understanding the organization of the brain and provide implications for clinical application. In the present study, we have demonstrated that the underlying structure of the lesion reflects the brain’s vasculature that can be utilized in lesion–symptom mapping to reduce mis-location error in stroke patients. In the future, one would need to formally test and validate the use of these PCA-derived lesion components in lesion–symptom mapping, prediction modelling and related neuropsychological approaches that aim to relate brain to behaviour.

## Supplementary Material

fcaa062_Supplementary_Data

## Abbreviations

BA =: Brodmann area
MCA =: middle cerebral artery
PCA =: principal component analysis
SCCAN =: sparse canonical correlation for neuroimaging
VBCM =: voxel-based correlational methodology
