# Supplementary material for: Evaluating the granularity and statistical structure of lesions and behaviour in post-stroke aphasia
Source: Brain Commun. 2020 May 19;2(2):fcaa062. doi: 10.1093/braincomms/fcaa062 (PMC7472896; doi:10.1093/braincomms/fcaa062)
Supplement: fcaa062_Supplementary_Data [file fcaa062_supplementary_data.zip › SupplementaryTable2-4.pdf]

Table 2. Lesion components location

	PCA Components	Size (voxels)	Percent On template	Peak Coordinates (MINI x, y, z)	Peak (*z*)
**Component 1**		**5191**		**-22 -6 12**	**9.20**
G	Putamen (L)	685	74%		
	Thalamus (L)	494	36%		
	Caudate (L)	373	66%		
	Insular Cortex (L)	252	22%		
	Pallidum (L)	186	60%		
W	Cortico Spinal (L)	1178	33%		
	Inferior Occipito Frontal Fasciculus (L)	455	33%		
	Corpus Callosum (L)	438	8%		
	Internal Capsule (L)	322	26%		
	Fornix (L)	284	23%		
	Uncinate (L)	198	22%		
**Component 2**		**4916**		**-64 -34 8**	**9.67**
G	Superior Temporal Gyrus, posterior division (L)	814	93%		
	Middle Temporal Gyrus, temporooccipital part (L)	560	66%		
	Middle Temporal Gyrus, posterior division (L)	505	40%		
	Planum Temporale (L)	405	76%		
	Angular Gyrus (L)	290	31%		
	Supramarginal Gyrus, posterior division (L)	285	27%		
	Heschls Gyrus (includes H1 and H2) (L)	208	65%		
	Lateral Occipital Cortex, superior division (L)	151	3%		
	Planum Polare (L)	107	30%		
	Superior Temporal Gyrus, anterior division (L)	102	40%		
W	Arcuate Posterior Segment (L)	243	33%		
	Inferior Longitudinal Fasciculus (L)	208	12%		
**Component 3**		**4946**		**-52 -2 40**	**-8.16**
G	Middle Frontal Gyrus (L)	1519	55%		
	Precentral Gyrus (L)	1367	32%		
	Inferior Frontal Gyrus, pars opercularis (L)	180	25%		
W	Corpus Callosum (L)	240	4%		
	Cortico Spinal (L)	229	7%		
**Component 4 Cluster 1**		**3043**		**-34 -48 10**	**-9.98**
W	Inferior Longitudinal Fasciculus (L)	610	35%		
	Arcuate Posterior Segment (L)	398	53%		
	Cortico Spinal (L)	359	10%		
	Optic Radiations (L)	310	57%		
	Corpus Callosum (L)	201	3%		
	Fornix (L)	151	12%		
	Inferior Occipito Frontal Fasciculus (L)	147	11%		
	Internal Capsule (L)	139	11%		
**Component 4 Cluster 2**		**1091**		**-30 -16 6**	**7.01**
G	Putamen (L)	420	46%		
	Pallidum (L)	147	47%		
	Insular Cortex (L)	123	11%		
W	Inferior Occipito Frontal Fasciculus (L)	128	9%		
**Component 5**		**3107**		**-14 -90 10**	**-5.10**
G	Lateral Occipital Cortex, inferior division (L)	788	39%		
	Occipital Fusiform Gyrus (L)	387	42%		
	Occipital Pole (L)	374	14%		
	Lingual Gyrus (L)	253	17%		
	Inferior Temporal Gyrus, temporooccipital part (L)	124	18%		
W	Inferior Occipito Frontal Fasciculus (L)	230	17%		
	Corpus Callosum (L)	206	4%		
**Component 6**		**4315**		**-40 -20 26**	**10.39**
G	Central Opercular Cortex (L)	681	69%		

	Postcentral Gyrus (L)	334	9%		
	Insular Cortex (L)	247	21%		
	Parietal Operculum Cortex (L)	129	22%		
	Precentral Gyrus (L)	122	3%		
W	Cortico Spinal (L)	710	20%		
	Arcuate Anterior Segment (L)	482	90%		
	Long Segment (L)	255	72%		
	Corpus Callosum (L)	121	2%		
**Component 7**		**3900**		**-30 44 -6**	**8.18**
G	Frontal Pole (L)	1129	16%		
	Frontal Orbital Cortex (L)	1078	63%		
	Inferior Frontal Gyrus, pars triangularis (L)	225	37%		
	Insular Cortex (L)	203	18%		
W	Corpus Callosum (L)	213	4%		
	Uncinate (L)	128	14%		
**Component 8**		**4718**		**-52 12 6**	**-8.71**
G	Precentral Gyrus (L)	649	15%		
	Inferior Frontal Gyrus, pars opercularis (L)	590	81%		
	Central Opercular Cortex (L)	418	42%		
	Frontal Operculum Cortex (L)	347	96%		
	Insular Cortex (L)	316	27%		
	Inferior Frontal Gyrus, pars triangularis (L)	249	41%		
W	Inferior Occipito Frontal Fasciculus (L)	137	10%		
	Arcuate Anterior Segment (L)	115	21%		
**Component 9 Cluster 1**		**3455**		**-66 -24 -14**	**-6.06**
G	Lateral Occipital Cortex, superior division (L)	923	19%		
	Middle Temporal Gyrus, posterior division (L)	588	46%		
	Lateral Occipital Cortex, inferior division (L)	514	25%		
	Inferior Temporal Gyrus, posterior division (L)	318	32%		
	Middle Temporal Gyrus, anterior division (L)	250	56%		
	Middle Temporal Gyrus, temporooccipital part (L)	235	28%		
	Inferior Temporal Gyrus, anterior division (L)	204	62%		
	Inferior Temporal Gyrus, temporooccipital part (L)	135	19%		
	Temporal Pole (L)	113	5%		
**Component 9 Cluster 2**		**597**		**-44 34 24**	**-5.66**
G	Middle Frontal Gyrus (L)	440	16%		
**Component 10 Cluster 1**		**2072**		**16 20 22**	**5.08**
W	Corpus Callosum (R)	442	8%		
	Internal Capsule (R)	299	13%		
**Component 10 Cluster 2**		**1637**		**-16 44 -10**	**4.99**
G	Frontal Pole (L)	473	7%		
	Putamen (L)	200	22%		
	Middle Frontal Gyrus (L)	153	6%		
	Frontal Orbital Cortex (L)	135	8%		
	Pallidum (L)	125	40%		
W	Anterior Commissure (L)	163	27%		
	Cortico Spinal (L)	110	3%		
	Uncinate (L)	106	12%		
**Component 11**		**5598**		**-16 -46 22**	**6.84**
W	Corpus Callosum (L)	1589	28%		
	Cingulum (L)	1187	24%		
	Cortico Spinal (L)	611	17%		
	Corpus Callosum (R)	222	4%		
	Cingulum (R)	100	2%		
**Component 12**		**5530**		**-18 -52 50**	**7.33**
G	Postcentral Gyrus (L)	1556	43%		
	Superior Parietal Lobule (L)	904	62%		
	Precentral Gyrus (L)	635	15%		
	Precuneous Cortex (L)	135	5%		

	Supramarginal Gyrus, posterior division (L)	108	10%		
W	Cortico Spinal (L)	301	9%		
	Corpus Callosum (L)	275	5%		
	Cingulum (L)	212	4%		
**Component 13**		**999**		**-2 10 38**	**5.54**
G	Paracingulate Gyrus (R)	221	16%		
	Cingulate Gyrus, anterior division (R)	183	14%		
	Cingulate Gyrus, anterior division (L)	104	10%		
**Component 14**		**4629**		**-56 -48 36**	**9.98**
G	Supramarginal Gyrus, anterior division (L)	685	72%		
	Supramarginal Gyrus, posterior division (L)	671	64%		
	Angular Gyrus (L)	548	58%		
	Parietal Operculum Cortex (L)	495	86%		
	Planum Temporale (L)	229	43%		
	Lateral Occipital Cortex, superior division (L)	131	3%		
W	Arcuate Posterior Segment (L)	441	59%		
	Cortico Spinal (L)	196	6%		
	Optic Radiations (L)	109	20%		
**Component 15**		**4537**		**-36 0 -30**	**9.34**
G	Temporal Pole (L)	1420	60%		
	Amygdala (L)	198	56%		
	Hippocampus (L)	150	18%		
	Middle Temporal Gyrus, anterior division (L)	147	33%		
	Pallidum (L)	137	44%		
	Planum Polare (L)	137	38%		
W	Inferior Longitudinal Fasciculus (L)	996	58%		
	Uncinate (L)	372	41%		
	Fornix (L)	189	15%		
	Anterior Commissure (L)	171	28%		
	Inferior Occipito Frontal Fasciculus (L)	156	11%		
	Corpus Callosum (L)	109	2%		
	Cortico Spinal (L)	106	3%		
**Component 16**		**5468**		**-24 -8 60**	**-6.12**
G	Superior Frontal Gyrus (L)	1678	66%		
	Paracingulate Gyrus (L)	554	41%		
	Middle Frontal Gyrus (L)	274	10%		
	Cingulate Gyrus, anterior division (L)	246	25%		
	Juxtapositional Lobule Cortex (Supplementary Motor Cortex) (L)	198	31%		
	Precentral Gyrus (L)	187	4%		
	Cingulate Gyrus, anterior division (R)	149	12%		
W	Cingulum (R)	521	12%		
	Corpus Callosum (R)	497	9%		
	Cingulum (L)	479	10%		
	Corpus Callosum (L)	414	7%		
**Component 17**		**2386**		**-62 -18 24**	**10.51**
G	Postcentral Gyrus (L)	881	24%		
	Precentral Gyrus (L)	688	16%		
	Supramarginal Gyrus, anterior division (L)	376	40%		
	Central Opercular Cortex (L)	222	23%		
	Inferior Frontal Gyrus, pars opercularis (L)	105	14%		
**Component 18**		**1461**		**34 -44 22**	**5.89**
W	Corpus Callosum (R)	257	5%		
	Arcuate Posterior Segment (R)	243	23%		
	Internal Capsule (R)	240	11%		
	Cortico Spinal (R)	214	8%		
	Inferior Longitudinal Fasciculus (R)	126	7%		
	Cingulum (R)	122	3%		
	Optic Radiations (R)	120	26%		
**Component 19 Cluster 1**		**941**		**-22 -74 16**	**5.36**

G	Lateral Occipital Cortex, superior division (L)	144	3%		
W	Corpus Callosum (L)	274	5%		
	Inferior Longitudinal Fasciculus (L)	200	12%		
**Component 19 Cluster 2**		**890**		**-22 -40 26**	**-6.31**
W	Cortico Spinal (L)	255	7%		
	Arcuate Posterior Segment (L)	181	24%		
	Corpus Callosum (L)	116	2%		
**Component 20 Cluster 1**		**1076**		**-20 -30 44**	**-6.02**
W	Cortico Spinal (L)	272	8%		
	Cingulum (L)	182	4%		
	Corpus Callosum (L)	148	3%		
**Component 20 Cluster 2**		**990**		**-30 -68 12**	**6.88**
W	Inferior Longitudinal Fasciculus (L)	257	15%		
	Corpus Callosum (L)	211	4%		
	Optic Radiations (L)	128	23%		
**Component 20 Cluster 3**		**778**		**-28 4 36**	**5.84**
W	Corpus Callosum (L)	182	3%		
	Cortico Spinal (L)	134	4%		

G: grey matter; W: white matter. Coefficents of each component were z-transformed and threshold at $|Z| > 3.29$ ($p < 0.001$). Clusters larger than 500 voxels were reported. For each cluster, detail regions were reported if it overlapped with more than 100 voxels of a specific region or white matter tract on the templates. We adopted the Harvard-Oxford cortical and subcortical templates, and natbrainlab white matter template. Percentage = significant voxels of this region / total voxels of this region based on the template.

Table 3. Voxel based correlation methodology with behaviours factors region reports

	Clusters for Behaviour Factors	Size (voxels)	Percent	Peak Coordinates (MINI x, y, z)	Peak (*r*)
**Phonology**					
	**Cluster 1**	**5641**		**-64 -18 -12**	**0.55**
**G**	Superior Temporal Gyrus, posterior division (L)	637	73%		
	Middle Temporal Gyrus, posterior division (L)	493	39%		
	Supramarginal Gyrus, posterior division (L)	439	42%		
	Planum Temporale (L)	296	56%		
	Planum Polare (L)	236	66%		
	Middle Temporal Gyrus, anterior division (L)	181	41%		
	Heschls Gyrus (includes H1 and H2) (L)	177	55%		
	Insular Cortex (L)	120	10%		
	Central Opercular Cortex (L)	93	9%		
	Middle Temporal Gyrus, temporooccipital part (L)	86	10%		
	Angular Gyrus (L)	65	7%		
	Parietal Operculum Cortex (L)	46	8%		
	Superior Temporal Gyrus, anterior division (L)	35	14%		
**W**	Inferior Longitudinal Fasciculus (L)	634	37%		
	Arcuate Posterior Segment (L)	547	74%		
	Optic Radiations (L)	198	36%		
	Inferior Occipito Frontal Fasciculus (L)	124	9%		
	Cortico Spinal (L)	114	3%		
	Long Segment (L)	88	25%		
	Internal Capsule (L)	65	5%		
	Corpus Callosum (L)	59	1%		
	Arcuate Anterior Segment (L)	53	10%		
	Fornix (L)	47	4%		
	Cortico Ponto Cerebellum (L)	28	3%		
**Semantics**					
	**Cluster 1**	**1175**		**-36 -12 -18**	**0.54**
**G**	Hippocampus (L)	133	16%		
	Amygdala (L)	74	21%		
	Temporal Pole (L)	32	1%		
	Temporal Fusiform Cortex, posterior division (L)	30	3%		
	Middle Temporal Gyrus, anterior division (L)	26	6%		
**W**	Inferior Longitudinal Fasciculus (L)	454	26%		
	Fornix (L)	130	11%		
	Uncinate (L)	104	11%		
	Inferior Occipito Frontal Fasciculus (L)	88	6%		
	Anterior Commissure (L)	42	7%		
	Optic Radiations (L)	37	7%		
	**Cluster 2**	**509**		**-34 -66 18**	**0.46**
**G**	Lateral Occipital Cortex, superior division (L)	83	2%		
**W**	Inferior Longitudinal Fasciculus (L)	169	10%		
	Optic Radiations (L)	109	20%		
	Cortico Spinal (L)	27	1%		
**Fluency**					
	**Cluster 1**	**9479**		**-60 -6 20**	**0.57**
**G**	Precentral Gyrus (L)	1537	36%		
	Postcentral Gyrus (L)	786	22%		
	Thalamus (L)	542	39%		
	Central Opercular Cortex (L)	342	35%		
	Paracingulate Gyrus (L)	282	21%		
	Caudate (L)	192	34%		
	Brain-Stem (L)	188	9%		
	Cingulate Gyrus, anterior division (L)	136	14%		
	Middle Frontal Gyrus (L)	111	4%		
	Juxtapositional Lobule Cortex (Supplementary Motor Cortex) (L)	66	10%		
	Pallidum (L)	56	18%		
	Hippocampus (L)	48	6%		

	Putamen (L)	32	3%
	Frontal Pole (L)	31	0%
	Inferior Frontal Gyrus, pars opercularis (L)	31	4%
	Cingulate Gyrus, posterior division (L)	23	2%
**W**	Cortico Spinal (L)	1444	41%
	Corpus Callosum (L)	839	15%
	Cingulum (L)	674	14%
	Internal Capsule (L)	453	37%
	Arcuate Anterior Segment (L)	292	54%
	Long Segment (L)	197	56%
	Fornix (L)	176	14%
	Superior Cerebelar Pedunculus (L)	66	9%
	Cortico Ponto Cerebellum (L)	55	7%
	Anterior Commissure (L)	52	9%

G: grey matter; W: white matter. Clusters were significant at Alphasim corrected $p < 0.01$ with voxel $p < 0.001$. For each cluster, detail regions were reported if it overlapped with more than 20 voxels of a specific region or white matter tract on the templates. We adopted the Harvard-Oxford cortical and subcortical templates, and natbrainlab white matter template. Percentage = significant voxels of this region / total voxels of this region based on the template.

Table 4. Regression analysis region reports

	Clusters for Behaviour Factors	Size (voxels)	Percent	Peak Coordinates (MINI x, y, z)	Peak (*z*)
**Phonology**					
	**Cluster 1**	**2546**		**-40 -42 2**	**8.12**
**W**	Inferior Longitudinal Fasciculus (L)	589	34%		
	Arcuate Posterior Segment (L)	387	52%		
	Optic Radiations (L)	300	55%		
	Cortico Spinal (L)	243	7%		
	Inferior Occipito Frontal Fasciculus (L)	143	10%		
	Internal Capsule (L)	117	9%		
	Corpus Callosum (L)	84	1%		
	Arcuate Anterior Segment (L)	77	14%		
	Long Segment (L)	76	22%		
	Fornix (L)	68	6%		
	Cortico Ponto Cerebellum (L)	43	5%		
	**Cluster 2**	**1393**		**-40 32 4**	**6.29**
**G**	Inferior Frontal Gyrus, pars opercularis (L)	239	33%		
	Frontal Operculum Cortex (L)	156	43%		
	Central Opercular Cortex (L)	131	13%		
	Inferior Frontal Gyrus, pars triangularis (L)	100	16%		
	Precentral Gyrus (L)	90	2%		
	Insular Cortex (L)	22	2%		
**W**	Inferior Occipito Frontal Fasciculus (L)	131	10%		
	Uncinate (L)	59	6%		
**Semantics**					
	**Cluster 1**	**2577**		**-32 -2 -30**	**5.73**
**G**	Temporal Pole (L)	464	20%		
	Amygdala (L)	156	44%		
	Putamen (L)	132	14%		
	Hippocampus (L)	118	14%		
	Pallidum (L)	102	33%		
	Thalamus (L)	54	4%		
	Temporal Fusiform Cortex, posterior division (L)	42	5%		
	Middle Temporal Gyrus, anterior division (L)	27	6%		
	Inferior Temporal Gyrus, anterior division (L)	23	7%		
**W**	Inferior Longitudinal Fasciculus (L)	651	38%		
	Uncinate (L)	223	24%		
	Fornix (L)	204	17%		
	Anterior Commissure (L)	162	27%		
	Inferior Occipito Frontal Fasciculus (L)	124	9%		
	Optic Radiations (L)	99	18%		
	Cortico Spinal (L)	97	3%		
	Internal Capsule (L)	64	5%		
	Superior Cerebelar Peduncululus (L)	24	3%		
	**Cluster 2**	**1117**		**-58 -38 40**	**7.18**
**G**	Supramarginal Gyrus, anterior division (L)	353	37%		
	Supramarginal Gyrus, posterior division (L)	332	32%		
	Parietal Operculum Cortex (L)	210	36%		
	Angular Gyrus (L)	176	19%		
**W**	Arcuate Posterior Segment (L)	35	5%		
	**Cluster 3**	**396**		**-32 -62 32**	**5.92**
**W**	Optic Radiations (L)	63	11%		
	Corpus Callosum (L)	53	1%		
	Inferior Longitudinal Fasciculus (L)	41	2%		
**Executive**					
	**Cluster 1**	**2434**		**-44 -78 20**	**5.66**
**G**	Lateral Occipital Cortex, superior division (L)	636	13%		
	Lateral Occipital Cortex, inferior division (L)	481	24%		
	Middle Temporal Gyrus, posterior division (L)	369	29%		
	Inferior Temporal Gyrus, posterior division (L)	199	20%		

	Inferior Temporal Gyrus, anterior division (L)	167	51%		
	Middle Temporal Gyrus, temporooccipital part (L)	126	15%		
	Temporal Pole (L)	90	4%		
	Middle Temporal Gyrus, anterior division (L)	89	20%		
	Inferior Temporal Gyrus, temporooccipital part (L)	60	9%		
**W**	Inferior Longitudinal Fasciculus (L)	50	3%		
	Optic Radiations (L)	34	6%		
	Cortico Spinal (L)	22	1%		
	**Cluster 2**	**600**		**-44 34 24**	**6.80**
**G**	Middle Frontal Gyrus (L)	390	14%		
	Frontal Pole (L)	126	2%		
	Inferior Frontal Gyrus, pars triangularis (L)	75	12%		
**Fluency**					
	**Cluster 1**	**3306**		**-52 -2 40**	**6.87**
**G**	Middle Frontal Gyrus (L)	1059	38%		
	Precentral Gyrus (L)	1001	23%		
	Inferior Frontal Gyrus, pars opercularis (L)	82	11%		
	Postcentral Gyrus (L)	40	1%		
**W**	Corpus Callosum (L)	184	3%		
	Cortico Spinal (L)	116	3%		
	Cingulum (L)	56	1%		
	**Cluster 2**	**2547**		**-22 -6 12**	**7.95**
**G**	Putamen (L)	425	46%		
	Thalamus (L)	336	24%		
	Caudate (L)	175	31%		
	Pallidum (L)	136	44%		
**W**	Cortico Spinal (L)	851	24%		
	Internal Capsule (L)	244	20%		
	Inferior Occipito Frontal Fasciculus (L)	155	11%		
	Fornix (L)	139	11%		
	Corpus Callosum (L)	47	1%		

G: grey matter; W: white matter. Behavioural voxel coefficients = stepwise regression model coefficients × principal component analysis coefficients. Then coefficients were z-transformed and thresholded at $|Z| > 3.29$ ($p < 0.001$). Clusters larger than 200 voxels were reported. For each cluster, detail regions were reported if it overlapped with more than 20 voxels of a specific region or white matter tract on the templates. We adopted the Harvard-Oxford cortical and subcortical templates, and natbrainlab white matter template. Percentage = significant voxels of this region / total voxels of this region based on the template.
