# Supplementary material for: Evaluating the granularity and statistical structure of lesions and behaviour in post-stroke aphasia
Source: Brain Commun. 2020 May 19;2(2):fcaa062. doi: 10.1093/braincomms/fcaa062 (PMC7472896; doi:10.1093/braincomms/fcaa062)
Supplement: fcaa062_Supplementary_Data [file fcaa062_supplementary_data.zip › SupplementaryFigs1-5.pdf]

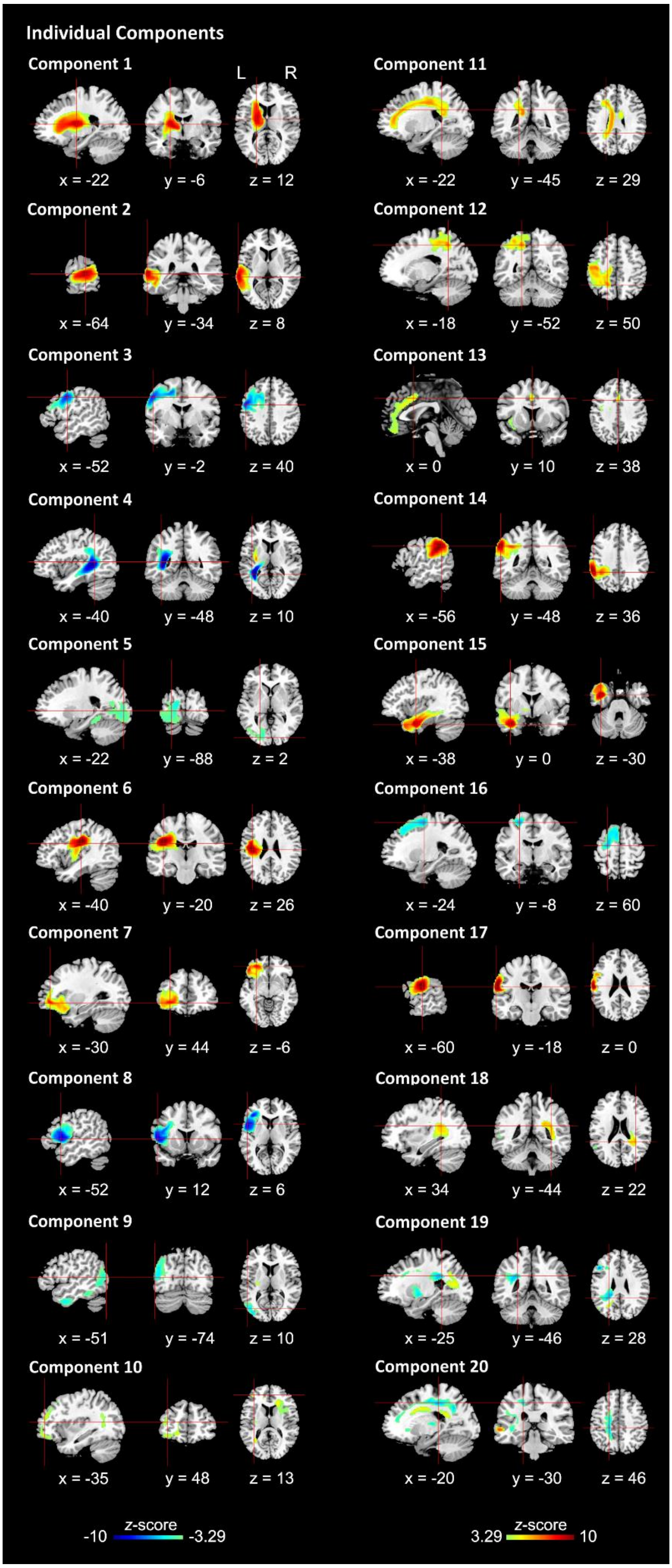

Supplementary Fig. 1. Principal components analysis (PCA) with 20 components based on the minimum description length. Each brain map displays the component weights thresholded at Z ± 3.29 (sign does not impact interpretation).

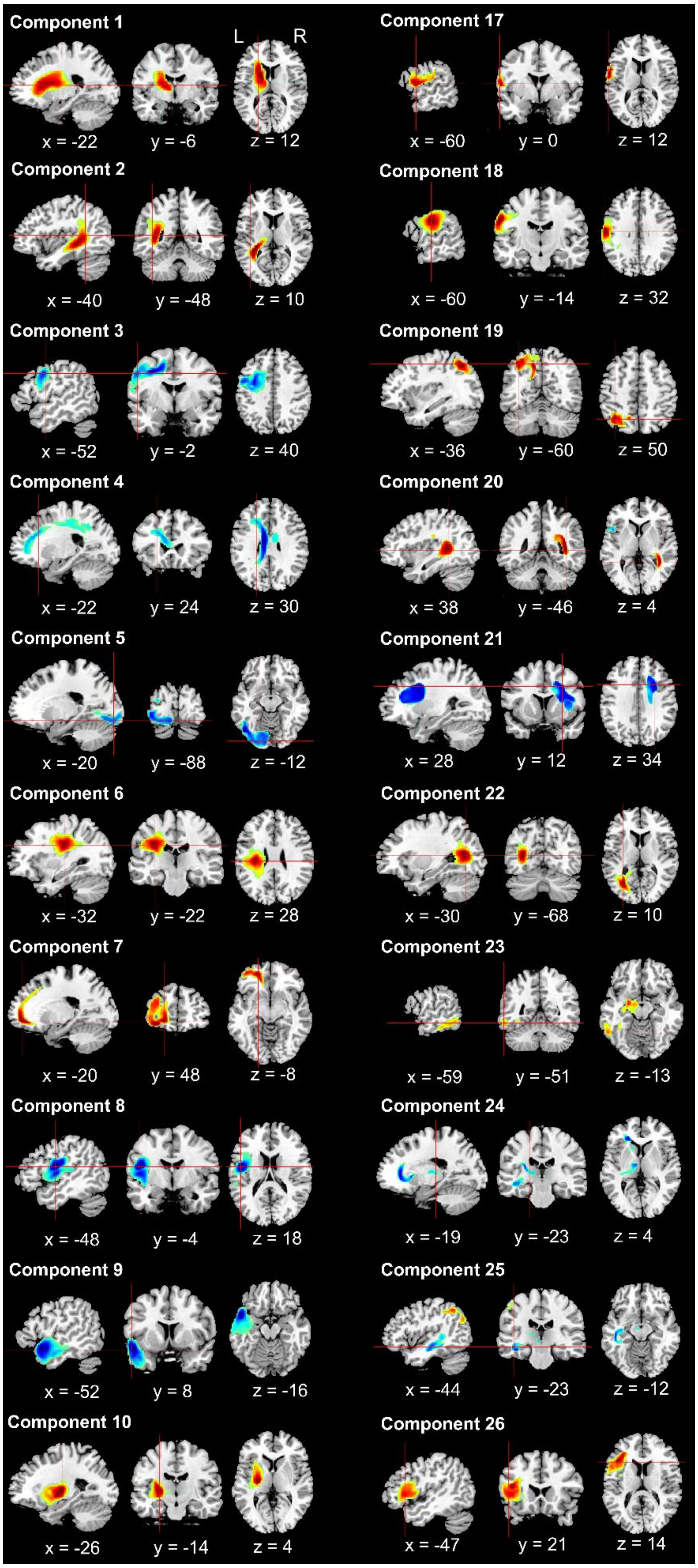

Component 1
L
R
x = -22
y = -6
z = 12
Component 2
x = -40
y = -48
z = 10
Component 3
x = -52
y = -2
z = 40
Component 4
x = -22
y = 24
z = 30
Component 5
x = -20
y = -88
z = -12
Component 6
x = -32
y = -22
z = 28
Component 7
x = -20
y = 48
z = -8
Component 8
x = -48
y = -4
z = 18
Component 9
x = -52
y = 8
z = -16
Component 10
x = -26
y = -14
z = 4
Component 17
x = -60
y = 0
z = 12
Component 18
x = -60
y = -14
z = 32
Component 19
x = -36
y = -60
z = 50
Component 20
x = 38
y = -46
z = 4
Component 21
x = 28
y = 12
z = 34
Component 22
x = -30
y = -68
z = 10
Component 23
x = -59
y = -51
z = -13
Component 24
x = -19
y = -23
z = 4
Component 25
x = -44
y = -23
z = -12
Component 26
x = -47
y = 21
z = 14

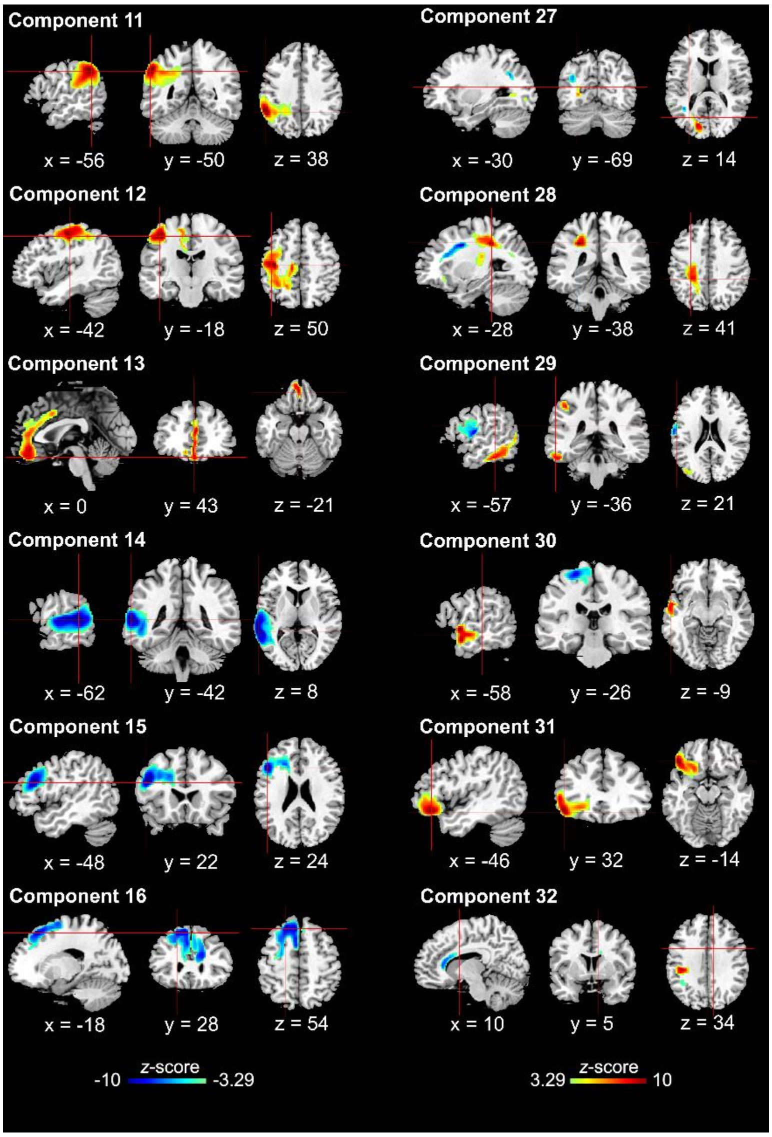

Supplementary Fig. 2. Principal components analysis (PCA) with 32 components based on Kolmogorov information criterion. Overall, the components match a 20 component model (see Supplementary Fig. 1) but certain larger components are now split into smaller clusters and a higher number of scattered components.

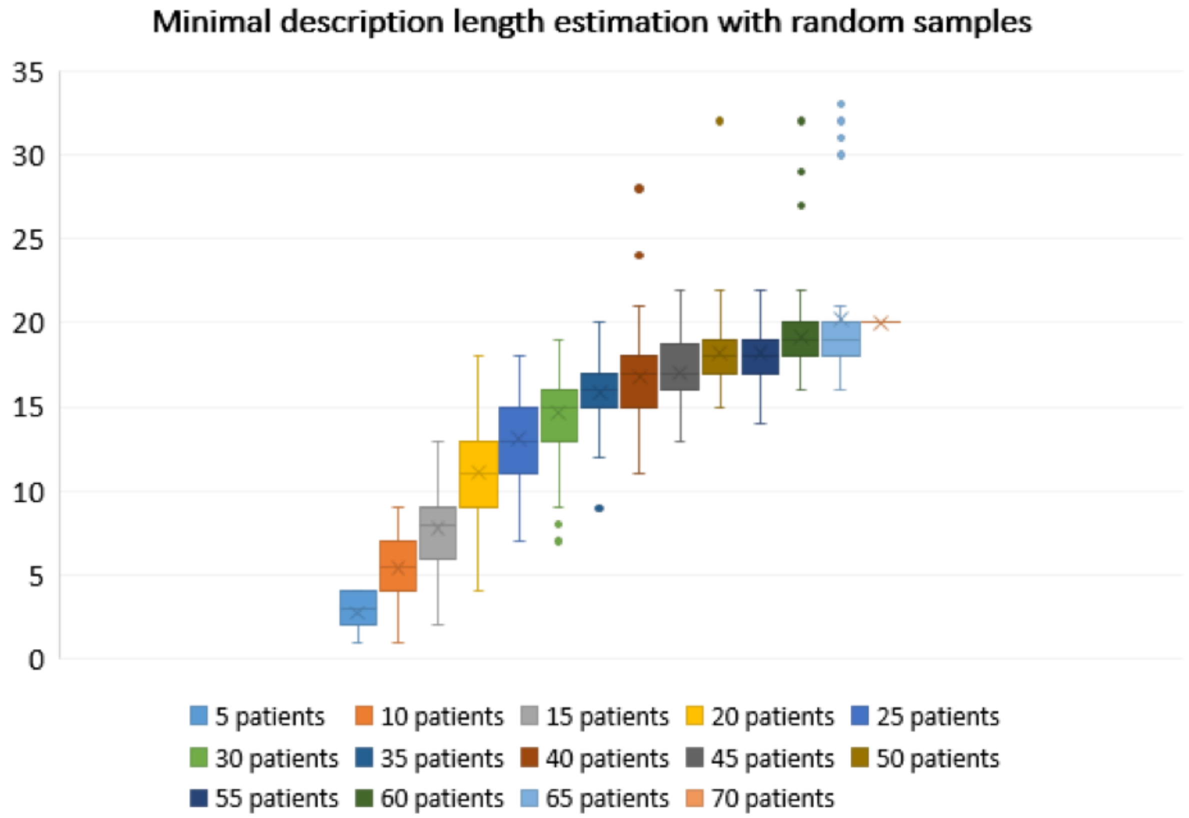

Supplementary Fig. 3. Minimal description length (MDL) estimation with random sub-samples. The vertical axis indicates the number of components suggested by MDL, while the horizontal axis indicates the sample size. The middle line of the box represents the median and the 'x' represents the mean. A data point is considered an outlier if it exceeds a distance of 1.5 times the first or third interquartile range. The MDL was repeated 100 times for each sub-sample out of a total sample of 70 patients.

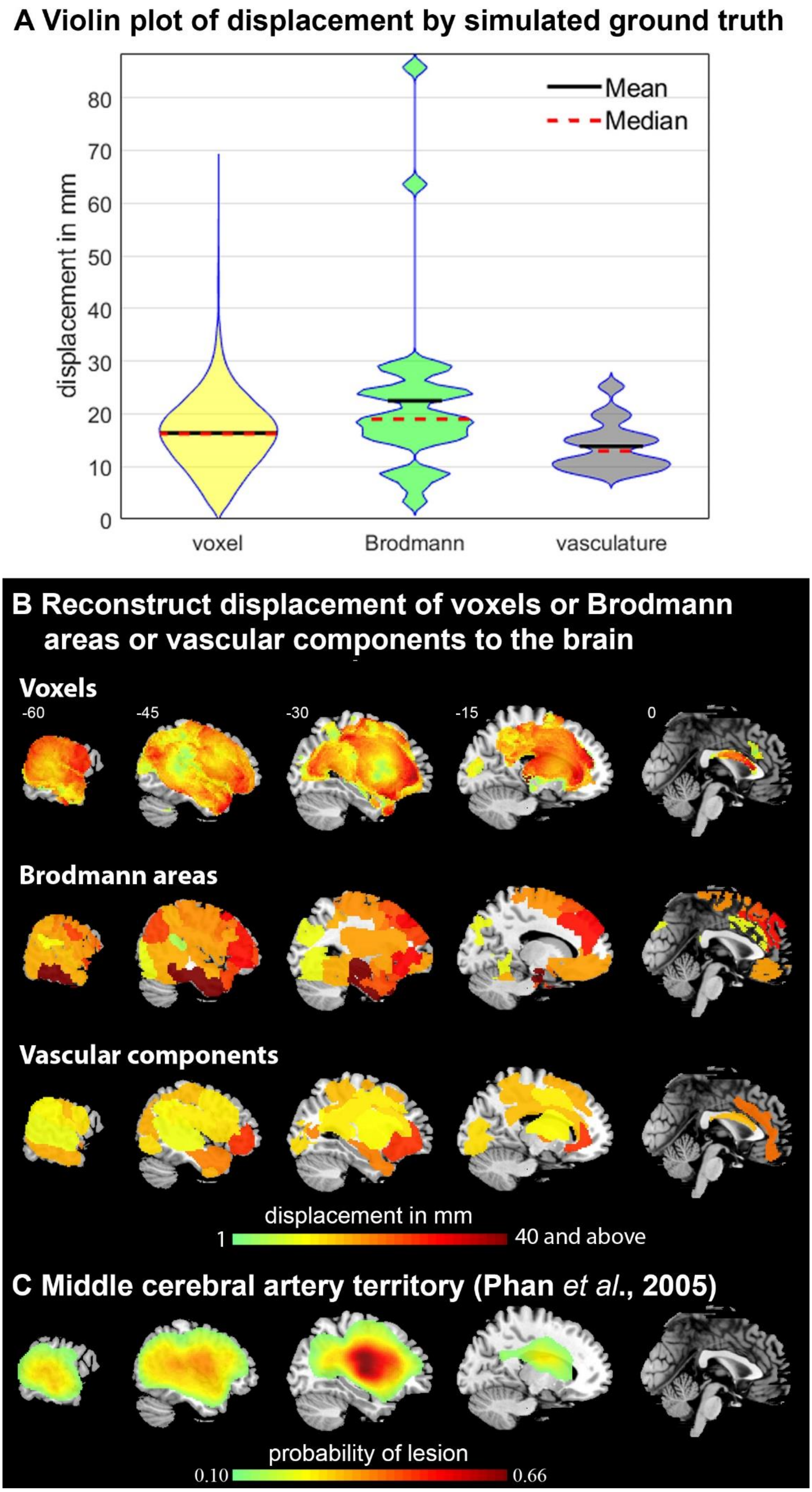

Supplementary Fig. 4. Displacement of centre of mass from simulated ground truths with different brain parcellations. At the group level, the simulated ground truth with vascular territories had significantly smaller displacement than simulations with voxels or Brodmann areas. Voxels or regions at the edges of the middle cerebral artery territory generally had the largest displacement.

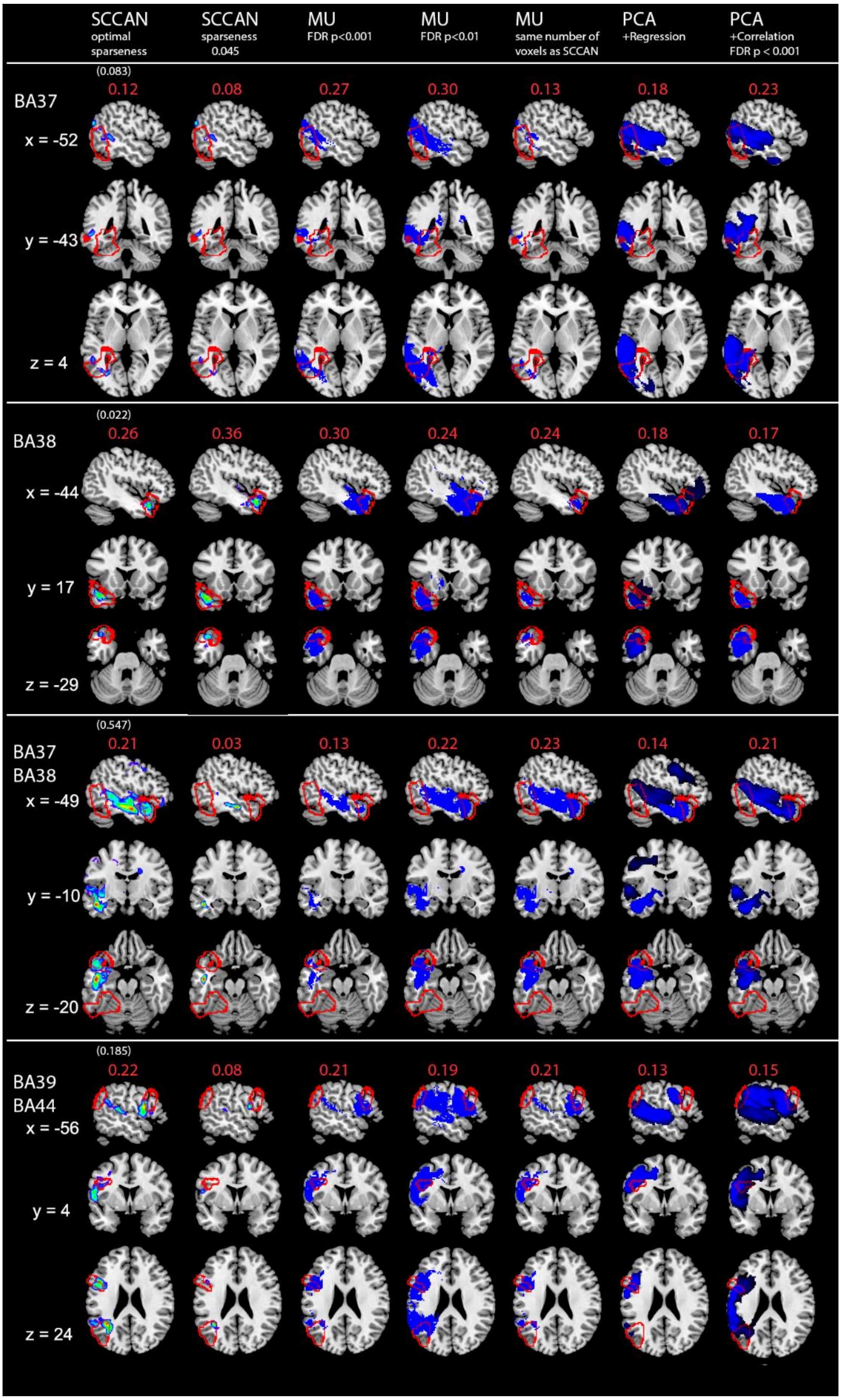

Supplementary Fig. 5. Comparing lesion-symptom mapping methods. Four exemplary ground truths were simulated (the red contours on the brain maps). Sparse canonical correlation for neuroimaging (SCCAN, Pustina et al., 2018), mass univariate analysis (MU), and principal component analysis (PCA) plus regression or correlation were used to make the spatial inference. For SCCAN with the optimal sparseness, the optimal sparseness values were given above the brain maps in white text. The Dice similarity coefficient between the ground truth and brain mapping result is given in red text above each brain map. The Dice coefficients do not identify a preference of one method over the others.
